# Prediction of Clinical Deep Brain Stimulation Target for Essential Tremor From 1.5 Tesla MRI Anatomical Landmarks

**DOI:** 10.3389/fneur.2021.620360

**Published:** 2021-10-27

**Authors:** Julien Engelhardt, Emmanuel Cuny, Dominique Guehl, Pierre Burbaud, Nathalie Damon-Perrière, Camille Dallies-Labourdette, Juliette Thomas, Olivier Branchard, Louise-Amélie Schmitt, Narimane Gassa, Nejib Zemzemi

**Affiliations:** ^1^Department of Neurosurgery, University Hospital of Bordeaux, Bordeaux, France; ^2^Institute for Neurodegenerative Disorders, CNRS–University of Bordeaux, Bordeaux, France; ^3^Department of Neurology, University Hospital of Bordeaux, Bordeaux, France; ^4^INRIA Bordeaux Sud-Ouest Research Centre, Talence, France; ^5^Mathematical Institute of Bordeaux, University of Bordeaux, Bordeaux, France

**Keywords:** essential tremor, Vim nucleus, deep brain stimulation (DBS) surgery, brain surgery, neurosurgery, machine learning

## Abstract

**Background:** Deep brain stimulation is an efficacious treatment for refractory essential tremor, though targeting the intra-thalamic nuclei remains challenging.

**Objectives:** We sought to develop an inverse approach to retrieve the position of the leads in a cohort of patients operated on with optimal clinical outcomes from anatomical landmarks identifiable by 1.5 Tesla magnetic resonance imaging.

**Methods:** The learning database included clinical outcomes and post-operative imaging from which the coordinates of the active contacts and those of anatomical landmarks were extracted. We used machine learning regression methods to build three different prediction models. External validation was performed according to a leave-one-out cross-validation.

**Results:** Fifteen patients (29 leads) were included, with a median tremor improvement of 72% on the Fahn–Tolosa–Marin scale. Kernel ridge regression, deep neural networks, and support vector regression (SVR) were used. SVR gave the best results with a mean error of 1.33 ± 1.64 mm between the predicted target and the active contact position.

**Conclusion:** We report an original method for the targeting in deep brain stimulation for essential tremor based on patients' radio-anatomical features. This approach will be tested in a prospective clinical trial.

## Introduction

Deep brain stimulation (DBS) of the ventro-intermedius nucleus of the thalamus (Vim) is a highly efficacious procedure to treat refractory essential tremor [ET] (1). Targeting the Vim, however, remains challenging. Surgery typically consists of an awake procedure with micro-electrode recordings (MER) and/or clinical testing to ensure lead positioning, despite its potential complications [intra-cerebral hematoma (2)] and disadvantages (length of the procedure, availability of suitably trained staff, and discomfort for the patient). Since DBS surgery for Parkinson's disease and dystonia are often performed under general anesthesia, there is also growing interest for asleep DBS for essential tremor. However, unlike the Globus Pallidus internus (Gpi) and subthalamic nucleus (STN), direct anatomical visualization of intra-thalamic nuclei remains technically challenging (3).

Asleep DBS for essential tremor with indirect targeting based on stereotactic coordinates has been described by Chen et al. with functional outcomes in the same range as the standard awake procedure (4, 5), though no prospective randomized trials have been performed to confirm this result. Numerous other targeting methods have been developed for Vim-DBS, including direct visualization of the Vim on white matter attenuated inversion recovery (6) or white matter nulled MPRAGE (7) sequences, dento-rubro-thalamic tract tractography [DRTt] (8, 9), but still under the control of micro-electrode recordings. Improvements in medical image analysis have allowed the development of more accurate probabilistic atlases (10–13), but DBS targeting based exclusively on these methods has not been reported. Indeed, most DBS centers have not yet taken the step toward asleep surgery without electrophysiology.

Several publications have studied the relationship between lead location and functional outcome for essential tremor, defining statistically optimal coordinates, mainly based on the width of the third ventricle for laterality and the AC-PC distance (1, 12, 14–24) for anteroposterior coordinates. The expression of a target as a linear function of only one anatomical landmark along each axis, however, might not be sufficient to summarize the inter-individual variability of brain anatomy. In this study, using machine-learning algorithms, we defined a personalized functional targeting based on the relationship between anatomical structures surrounding the Vim and lead locations in a cohort of patients operated on for essential tremor with optimal outcomes.

## Materials and Methods

### Study Design

In this retrospective and prospective observational study (PROBA-VIM), we developed a reverse approach to allow the construction of a probabilistic functional target efficient on essential tremor. Then, we performed a retrospective study on the consecutive patients operated on in our institution with the PROBA-VIM target.

### Patients

All the consecutive patients who underwent VIM-DBS for ET between January 1, 2009 and June 1, 2018 (retrospective part) and after June 1, 2018 (prospective part) in our institution were considered for inclusion. Inclusion criteria were as follows: patient over the age of 18 years, having undergone DBS surgery targeting the Vim at Bordeaux University Hospital for the strict indication of essential tremor. Patients were excluded if they underwent VIM-DBS for different indications (Parkinson's disease for instance, in questionable cases, a SPECT with ^123^I-Ioflupane (DAT-scan) was performed to eliminate any dopaminergic denervation). This cohort of patients is named after “training set” in the paper.

After having developed the PROBA-VIM target with the training set cohort, we offered to all the consecutive patients suffering from severe essential tremor not controlled under medical treatment and who were not a candidate for an awake procedure with micro-electrode recordings because of their age, cerebral atrophy, or refusal, the possibility to be operated on without MER under general anesthesia with the PROBA-VIM targeting one step procedure. The patients were made aware of the experimental nature of the procedure, and therefore of the potential risk of failure. This cohort of patients is named after “validation set” in the paper.

### DBS Surgery

A Leksell frame (ELEKTA^TM^) was positioned under local anesthesia (lidocaine) and aT1 WI (SIEMENS ACHIEVA^TM^) was performed with the following parameters: 1.5 Tesla, TR = 15 ms, TE = 7 ms, matrix 512^*^512, flip angle 30°, band width 15 kHz and slice thickness 1 mm. The stereotactic coordinates used for Vim targeting were *x* = 13; *y* = −4; *z* = 0 with MCP at the origin. The five-microelectrode guide was inserted using the NEUROMATE^TM^ surgical robot under O-ARM © (MEDTRONIC^TM^) control. Electrophysiological recordings began 10 mm above the predefined target. Neuronal activity was stored at different levels with the neuronal discharge characteristics according to discharge frequency, in response to intra-operative cutaneous somaesthetic stimulation and rapid active and passive joint movements along with single unit activity pattern. Depending on the electrophysiology results, stepped stimulations using 3–5 electrodes were performed. These made it possible to quantify: (1) clinical efficacy on tremor and (2) possible side effects of stimulation. A 3D intra-operative O-ARM © scanner fused with the stereotactic MRI scan was used to calculate the frame-based coordinates of the electrophysiological target. The 3389 MEDTRONIC^TM^ electrode was then inserted to this defined target under O-ARM © control.

### Clinical Evaluation

#### Training Set

Evaluation of the patients in the training set was performed at least 3 months post-operatively. Efficacy of DBS on tremor was quantified by ON-stimulation and OFF-stimulation assessment 30 min after the DBS was turned off to eliminate any rebound effect. We used a limited version of the Fahn–Tolosa–Marin (FTM) scale [first 15 items because the last 6 items evaluate tremor consequences on daily living activities] (25).

An optimal post-operative result was defined by complete withdrawal of medication, stability of the stimulation effects without any setting adjustments since at least 3 months and an improvement of more than 66% on the limited FTM scale between OFF- and ON-stimulation assessments.

We assessed the efficacy of each individual lead contact on tremor as follows. Once stimulation was turned off, we increased the voltage in 0.1 Volt increments in monopolar stimulation until the appearance of permanent adverse effects. To standardize comparison between the different contacts of a given lead, we stimulated each contact with the same parameters (pulse width and frequency) used for chronic stimulation in this electrode. Tremor was assessed by items 5 and 6 of the FTM scale.

#### Validation Set

Evaluation of the patients further operated on with the PROBA-VIM target was performed at least 3 months post-operatively. Efficacy of the stimulation was quantified by the percentage of improvement between the pre-operative and the post-operative evaluations with the entire FTM scale.

### Lead Location in Stereotactic Space

Patients underwent either a post-operative 3D T1 MRI on a SIEMENS ACHIEVA^TM^ with the following parameters: 1.5 Tesla, SAR <0.1 W/kg, TR = 15 ms, TE = 7 ms, matrix 512^*^512, flip angle 30°, band width 15 kHz and slice thickness 1 mm or a post-operative CT scan at 1 mm slice thickness which was co-registered to the pre-operative MRI if a post-operative MRI was not available.

The DBS lead was traced on the post-operative imaging along the metallic artifact and reconstructed by using the coordinates of the distal tip of the lead (point A) and the coordinates of a point chosen just proximal to the last active contact (point J). The coordinates of the center of each contact were calculated according to the following formula giving the coordinates of any point “M” of the DBS lead:


$$\begin{array}{l} \left(\begin{array}{c} x_{M}\\ y_{M}\\ z_{M} \end{array}\right)=D.\left(\begin{array}{c} X_{u}\\ Y_{u}\\ Z_{u} \end{array}\right)+\;\left(\begin{array}{c} x_{A}\\ y_{A}\\ z_{A} \end{array}\right)\;, \end{array}$$

with D being the distance between A and M and $\vec{u}$ the unitary director vector of the lead, calculated from the coordinates of A and J, see reference (26) for more details. The data were systematically confirmed by manual analysis of the coordinates given by the middle of each hypo-intensity shadow representing each contact. In case of bipolar stimulation, the middle of the two contacts was considered as the target. Coordinates are given in the conventional (AC-PC) space, with PC at the origin. STEALTHSTATION S7 © (MEDTRONIC^TM^) was used for image processing.

The stereotactic accuracy of DBS-lead placement in the validation set was defined according to two parameters. Firstly, we calculated the distance (*d*_1_) between the center of the active contact (or the middle of the two active contacts) and the planned target as follows: $d_{1}=\;\|\vec{MT}\|$ with $\vec{MT}$ being the vector between the middle of the active contact (*M*) and the planned target (*T*). Secondly, we calculated the distance (*d*_2_) between the axis of the DBS lead and the planned target (*t*), which is the minimal distance between the target and the lead, as follows: $d_{2}=\;\frac{\left\|\vec{LT}\;ˆ\;\vec{u}\right\|}{\left\|\vec{u}\right\|}$ with $\vec{u}$ being the direction vector of the lead and $\vec{LT}$ the vector between the tip of the lead (*L*) and the planned target (*T*).

### Active Contact Locations on the DISTAL Atlas in MNI Space

Active contacts were normalized on the DISTAL atlas in MNI space [ICBM 2009b template] (11) then the DISTAL atlas was merged onto the MNI template to obtain the overlay of active contacts on the anatomical structures segmented according to the atlas. All these operations were carried out using the open-source Lead-DBS software © (www.lead-dbs.org) running on MATLAB © (MATHWORKS^TM^).

### Radio-Anatomical Landmarks

**Figure 1:**
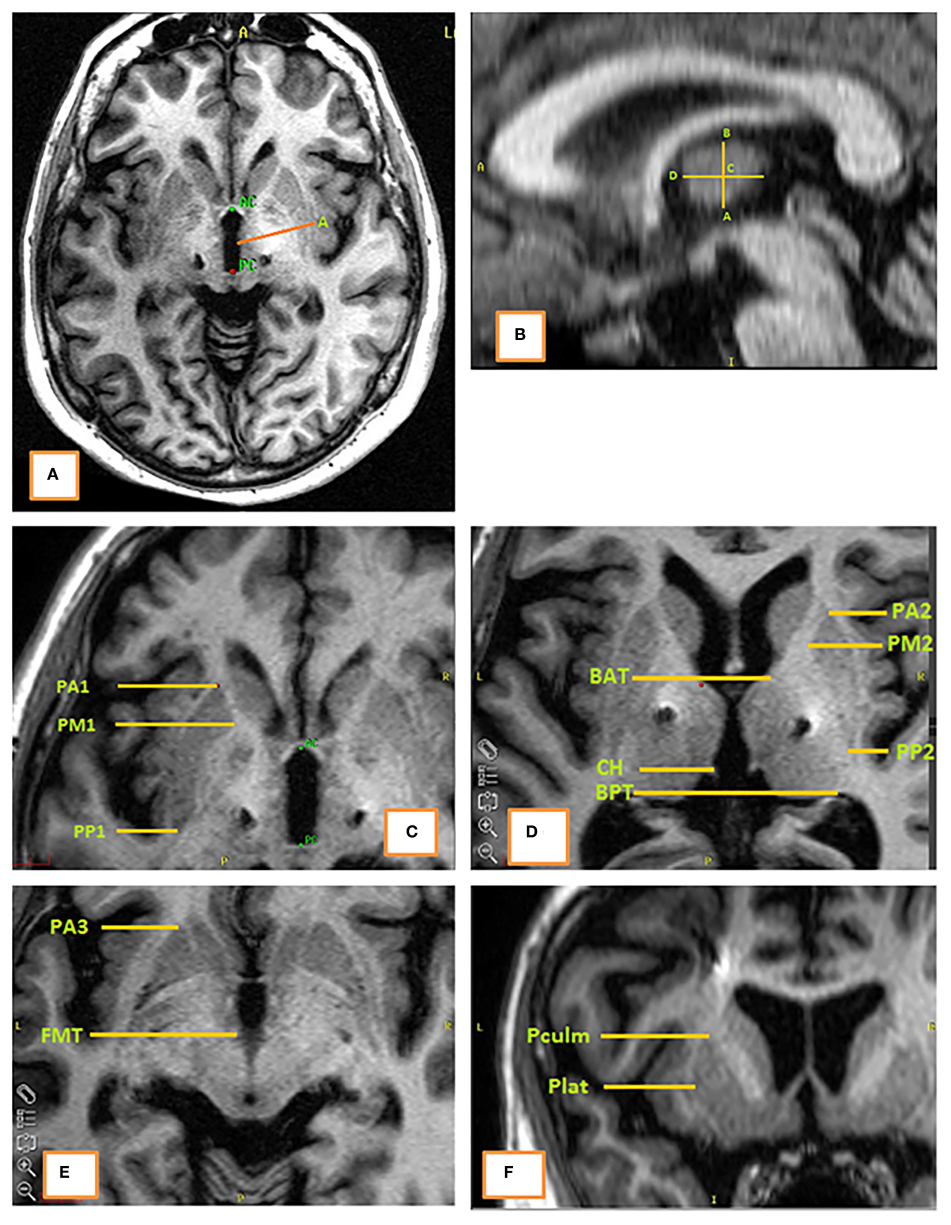
The 18 anatomical landmarks on a T1-weighted MRI. **(A)** Axial plane at the level of (AC-PC); **(B)** sagittal plane at the level of landmark “A” (lateral border of the third ventricle); **(C)** axial plane at the coordinate *z* = *Z*_C_ – 5 mm; **(D)** axial plane at the coordinate *z* = *Z*_C_; **(E)** axial plane at the coordinate *z* = *Z*_C_ – 10 mm; **(F)** coronal plane at the level of landmark “PM1.”

A multidimensional patient-specific referential was defined with 18 landmarks for each hemisphere (Figure 1). We selected anatomical structures close to the target (e.g., boundaries of the thalamus, putamen) that were readily identifiable by 1.5 Tesla T1-weighted magnetic resonance imaging without Gadolinium infusion. The objective was to have as many landmarks as possible to delimit a “bounding box” around the target area. Once this first step had been completed, the second step consisted in evaluating the inter- and intra-observer reproducibility of the positioning of these landmarks by the neurosurgeons. The third step consisted in selecting the most relevant landmarks, or by filtering the less relevant landmarks, by the learning algorithms. The 18 landmarks were:

- In the axial plane *z* = 0: AC (anterior commissure), A [lateral border of the third ventricle at y = MCP (mid-commissural point)]
- In the parasagittal plane x = X_A_: B (the highest point of thalamus at y = MCP), C (the middle of [AB], D (the most anterior point of the thalamus at z = Z_C_)
- In the axial plane z = Z_C_: PA2, PM2, PP2 (the anterior, median and posterior edges of the putamen, respectively), BAT and BPT (the most anterior and posterior points of the thalamus, respectively), and CH (habenular commissure)
- In the axial plane z = Z_C_ – 5: PA1, PM1, PP1 (the anterior, median and posterior edges of the putamen, respectively)
- In the axial plane z = Z_C_ – 10: PA3 (the anterior edge of the putamen), FMT (mammillothalamic tract)
- In the coronal plane y = Y_PM1_:Pculm and Plat (the highest and the more lateral points of the putamen, respectively)

## Modeling of the Optimal Target Prediction (Proba-VIM Target)

### General Description of the Methods

In this paragraph, we describe only the principles of the three machine-learning approaches used in this study: (i) reproducing kernel Hilbert space (RKHS): kernel ridge regression method (27); (ii) support vector regression (27–30) [SVR]; (iii) deep neural networks (31) [DNN]. The aim was to determine a function ***F*** that continuously maps the anatomical landmarks to active contact locations. In order to formalize the problem, we denoted by *X* the vector representing the landmarks. The vector *X* was obtained by concatenating the coordinates of the landmarks. Each vector *X* had a corresponding target (the active contact) which was denoted by *Y*. We constructed the matrix of (landmarks, target) pairs: (*X*_*i*_, *Y*_*i*_) *i* = 1,…, *N* for a population of *N* hemispheres extracted from the post-operative MRI data. The data (*X*_*i*_, *Y*_*i*_), *i* = 1,…, *N* were the training database.

The function that mapped the features to the target was called the metamodel. The exact expression of the function ***F*** depends on the chosen method. We, thus, had three different metamodels, *F*_*RKHS*_, *F*_*SVR*_, and *F*_*DNN*_.

### Kernel Ridge Regression Method (RKHS)

Following the RKHS kernel ridge regression method (27), we looked for the function *F* defined as follows:


(1) $$\begin{array}{l} F\left(x\right)=\overset{n}{\underset{i=1}{\sum }}\alpha_{i}\text{K}\left(x,{\text{X}}_{\text{i}}\right), \end{array}$$

where the coefficients α_*i*_ are to be determined and *K* is a Gaussian kernel $K\left(\text{x},\text{y}\right)=\;e^{-\frac{|\text{x}-\text{y}\;{|}^{2}}{{2\sigma }^{2}}}.$

The value of the parameters *σ*, and the coefficients *α*_*i*_, *i* = 1,…, *N* were obtained by minimizing the following least square function:


(2) $$\begin{array}{l} \left\{\frac{1}{n}\overset{\text{n}}{\underset{i\;=\;1}{\sum }}{\left(F\left(X_{\text{i}}\right)-\;{\text{Y}}_{\text{i}}\right)}^{2}+\;\lambda \|F{\|}_{H}^{2}\right\} \end{array}$$

with respect to the coefficients *α*_*i*_, *i* = 1,…, *N* and parameters σ and λ where λ is a regularization coefficient. Since the function F mapped the vector of the concatenated input landmarks to a 3D-coordinate position, the prediction of the active contact was performed coordinate by coordinate.

#### Deep Neural Network Method (DNN)

In order to describe the DNN method (31), we first needed to define some functions. Let *W*_*l*_ be the affine function representing the weights and bias for the first layer of the network and let *a*_*l*_ be the neurons in the first layer. The affine transformation from layer (l – 1) to layer l is given by:


$$\begin{array}{l} {\text{W}}_{\text{l}}\left({\text{a}}_{\text{l}-1}\right)=\;{\text{A}}_{\text{l }}{\text{a}}_{\text{l}-1}+b_{l}. \end{array}$$

Matrix A_l_ represents the weights; the number of its lines is equal to the size of the first layer and the number of its columns is equal to the size of the layer number (l – 1). Vector *b*_*l*_ is the bias of the first layer; its size is equal to the number of neurons in this layer. Note that *A*_*l*_ and *b*_*l*_ are determined during the training process.

We denoted by *f*_*l*_ the function that maps the neuros of layer (l – 1) to the neurons of layer l.


$$\begin{array}{l} {\text{f}}_{\text{l}}\left(a_{\text{l}-1}\right)=\text{ r}\left({\text{A}}_{l\;}a_{\text{l}-1}+\;{\text{b}}_{\text{l}}\;\right), \end{array}$$

where *r* is the rectified linear unit (RELU), *r*(*x*) = max(*x*, 0).

Defining L as the number of layers in the DNN, the metamodel we aim to build could be written as a composition of the described functions as follows (31):


$$\begin{array}{l} {\text{F}}_{\text{DNN}}\left(x\right)=\;{\text{f}}_{\text{L }}o\;{\text{f}}_{\left(L-1\right)}o\;{\text{f}}_{\left(L-2\right)}o\ldots o\;{\text{f}}_{2\;}o\;{\text{f}}_{1\;}\left(\text{x}\right). \end{array}$$

In practice, we used three hidden layers of sizes 256, 128, and 64, respectively (see Supplementary Figure 1). We used the TensorFlow library (32) to train our metamodel.

### Support Vector Regression (SVR)

The metamodel using the SVR method (33) is written as follows:


$$\begin{array}{l} {\text{F}}_{\text{SVR}}\left(\text{x}\right)=\sum_{i\;=\;1}^{\text{N}}\left(\alpha_{\text{i}}\;-\;\alpha_{\text{i}}^{*}\right)k\left(x,X_{\text{i}}\right)+b \end{array}$$

where *k* is a Gaussian kernel $K\left(\text{x},\text{y}\right)=\;e^{-\frac{|\text{x}-\text{y}{|}^{2}}{{2\sigma }^{2}}}$ and α_*i*_, $\alpha_{i}^{*,}$ and *b* are obtained by solving the following constrained minimization problem.


$$\underset{\zeta ,\zeta^{\text{*}},b,W}{\min}\frac{1}{2}{\left|\left|W\right|\right|}^{2}+C\sum_{i\;=\;1}^{N}(\zeta_{i}-\zeta_{i}^{*})$$

subject to $\left\{ \begin{array}{l} \;\left(<W\;.\;X_{i}>+b\right)-Y_{i}\leq \varepsilon +\;\zeta_{i},\\ Y_{i}-\;\left(<W\;.\;X_{i}>+b\right)\;\leq \varepsilon +\;\zeta_{i}^{*},\\ \zeta_{i},\zeta_{i}^{*}\;\geq 0,\;for\;i=1,\;\ldots N. \end{array}\right.$

where the parameters, ε, *C*, and σ, are optimized using a grid search. We used the scikit-learn library (34) to train our metamodel. In this method also, the prediction of the active contact was performed coordinate by coordinate in order to construct the 3D position of the predicted contact.

### Intra- and Inter-Observer Variability of Landmarks and Data Augmentation

To quantify the uncertainty of manual landmark segmentation, two senior neurosurgeons performed the segmentation of the 18 landmarks for the 15 MRI data considered in the study; and one senior neurosurgeon performed the segmentation twice. For each landmark, on each axis, we computed the Euclidian distances between segmentations. This allowed us to construct a Gaussian law of the inter- and intra-observer segmentation uncertainty for each landmark.

We used the landmark segmentation uncertainty to generate artificial data. This allowed us to determine whether data augmentation improved the performance of the prediction and, secondly, enabled us to quantify the propagation of uncertainty from the input data to the predicted targets. Consequently, we provided a target with a confidence interval.

### Metamodel Evaluation: External Validation by Cross-Validation and Lead Location on Atlas

We provided two approaches for evaluating the performance of the three methods. The first was based on cross-validation statistical testing. We measured the distance between the predicted and the clinical targets. In this approach, we removed one patient (both hemispheres) from the training set and compared the prediction calculated by the metamodels with the (*n* – 1) patients to the active contact (leave-one-out cross-validation, LOOCV). This step was performed iteratively for all the patients of the database. The distances (mean ± SD) between the predicted target and active contacts were then calculated.

In the second approach, we compared the position of the predicted target to the anatomical structure of the VIM from the DISTAL atlas (11) in MNI space.

### Clinical Evaluation of the Metamodel

The “validation set” cohort was used to illustrate the clinical outcomes of DBS surgery under general anesthesia without the MER procedure.

### Statistical Analysis

Quantitative variables were presented as mean with standard deviation (SD) or as median with interquartile range (IQR) depending on their distribution (tested by the Kolmogorov–Smirnov test) and were compared using the two-tailed Student's test (5% alpha level). Qualitative variables were presented as values (%). Differences were considered to be statistically significant if *p* < 0.05. SPSS STATISTICS v. 24 © (IBM^TM^) software was used.

### Ethical Approval

All patients were informed about the purpose of this study and informed consent was obtained from all participants and/or their legal guardians. The prospective part of this study was approved by the regional ethical committee (CPP OUEST IV) and is registered under NCT 03696420. The patients operated on with the PROBA-VIM target gave their informed consent and the analysis of their medical records was approved by the Institutional Review Board. All this research was conducted in accordance with relevant guidelines/regulations.

## Results

### Patient Characteristics of the Training and the Validation Sets

**Figure 2:**
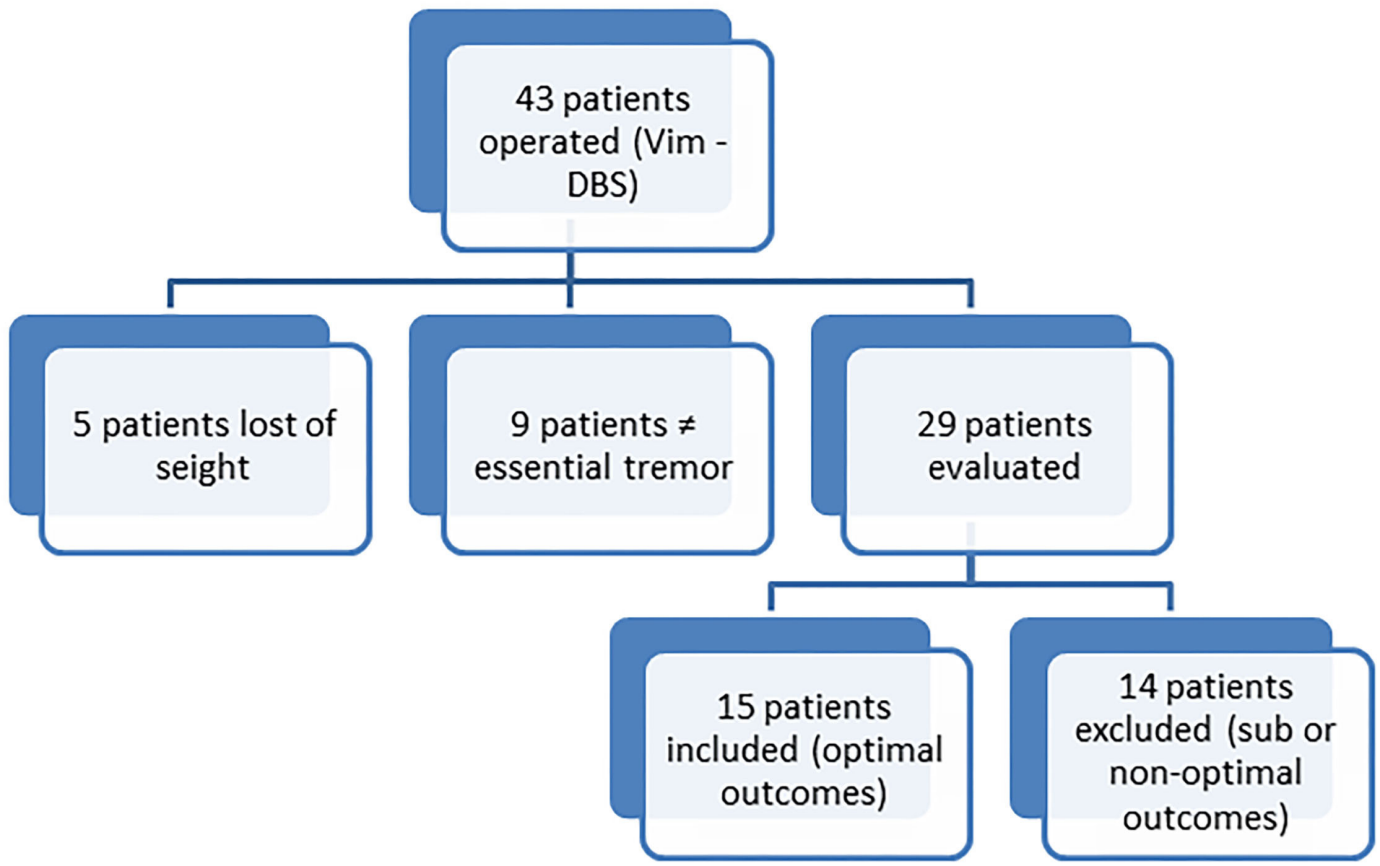
Flowchart showing the process of patient selection in order to construct the training database. The obtained training data base contains 15 patients.

Fifteen patients were included in the training set (see Flowchart, Figure 2). Demographic characteristics as well as outcome variables are detailed in Table 1.

**Table 1:** Patients' characteristics (training set).

Patients	15
Center side	15
Right side	14
Leads	29
Age at surgery	64 [60–67]
Sex ratio M:F	2.6
Time point of evaluation (months after surgery)	13 (8-27)
**Clinical outcome**	
15-item FTM “OFF stimulation”	38 (30-41)
15-item FTM “ON stimulation”	9 (6-12)
Improvement (%)	72 [67–82]
**Chronic stimulation settings**	
Amplitude (Volt)	2.2 [2–2.5]
Pulse width (μs)	60 [60–90]
Frequency (Hz)	130 [130–140]
**Active contact in patient's AC-PC space (origin = PC, mm)**	
*X*	14.1 [13.5; 14.8] min: 11.33/max: 18.20
*Y*	8.0 [7.1; 8.9] min: 2.86/max: 12.70
*Z*	0.7 [−0.4; 1.8] min: −3.20/max: 6.12
**Active contact in normalized MNI space**	
*X*	14.5 [13.8; 16.5] min: 11.9/max 19.5
*Y*	−16.4 [−18.1; −15.3] min: −20.5/max −6.6
*Z*	−3.58 [−5.7; 0.15] min: −11.0/max 9.21

Nine consecutive patients were operated on under general anesthesia without MER with the PROBA-VIM target (validation set). Demographic characteristics as well as outcome variables are detailed in Table 2.

**Table 2:** Patients' characteristics (validation set).

Patients	9
Center side	9
Right side	9
Leads	18
Age at surgery	71 [67–77]
Sex ratio M:F	2
Time point of evaluation (months after surgery)	14 (9-23)
**Clinical outcome**	
Pre-operative FTM	55 [53–58]
Post-operative FTM	20 (12-35)
Improvement (%)	64 (40-75)
**Chronic stimulation settings**	
Amplitude (Volt)	1.9 [1.5–2.5]
Pulse width (μs)	60 [60–82.5]
Frequency (Hz)	130 [130–130]
**Stereotactic accuracy**	
Distance between active contact and planned target (mm)	3.02 [2.35; 3.68] min: 1.50/max: 12.47
Distance between DBS-lead and planned target (mm)	1.41 [0.96; 2.12] min: 0.09/max: 8.54

### Active Contact Locations on the DISTAL Atlas in MNI Space

First, we observed a normalization defect that we could not correct on 3 electrodes (aberrant position with 1 contact located in the right cerebral peduncle and 2 in the corona radiata). Supplementary Table 1 summarizes the position of active contacts according to Hassler's nomenclature: one active contact (3%) was located in the Zce (zentrolateraliscaudalis pars externa); 8 active contacts (28%) in the Vim; 3 (10%) in the Voi (ventro-oralis internus); 14 (48%) in the ZI (zona incerta); and 3 (10%) were excluded because of an aberrant position.

**Figure 3:**
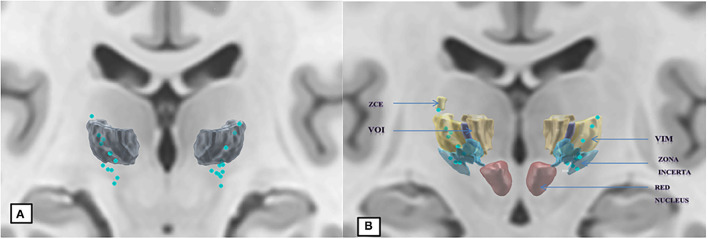
Normalization of 26 out of 29 active contacts, template ICBM2009b, atlas DISTAL. **(A)** Segmentation of the Vim. **(B)** Addition of adjacent structures according to Hassler's nomenclature.

According to this method (Figure 3), we observed that the active contacts were schematically located in two different structures: the conventional intra-thalamic target (38%) which includes the Vim and Voi nuclei [the ventral lateral posterior nucleus—VLp—in Hirai and Jones nomenclature, consisting of the cerebellar territory of the motor thalamus (35)], and a subthalamic “zone” (48%). Note that the zona incerta is very extensive in the DISTAL atlas used (Figure 3B), and most certainly includes the Forel H1 field. It is very likely that the active contacts were in fact located in this site, where the dento-rubro-thalamic tract runs. It should be noted that an active contact was located very high in the thalamus, in the Zce nucleus which is not known to receive cerebellar afferences, or to present a cytoarchitectonic organization comparable to Vim (36). We could have also considered this contact to be in an aberrant position; however, we did not notice any abnormalities in either the electrode reconstruction or imaging normalization stage for this patient.

### Intra- and Inter-Observer Variability in Landmark Positioning

The mean intra- and inter-observer variability values for landmark locations were 0.52 ± 0.37 and 0.60 ± 0.59 mm with maximum values obtained for X_BPT_ with 1.70 and 4.14 mm, respectively. Detailed information is provided in Supplementary Material. The mean intra- and inter-observer variability values for active contact locations were 0.44 ± 0.18 and 0.27 ± 0.06 mm, respectively.

### Landmark Selection

The only method for which we performed feature selection was RKHS. Selected features were: BAT x-axis, BAT y-axis, BPT x-axis, GPE PA2 y-axis, GPE PA3 x-axis, FMT x-axis, GPE Pculm Z-axis, and GPE Plat Z-axis.

The SVR method performed the selection on the patients themselves because the support vectors correspond to the landmarks of a subset of patients. The DNN method is assumed to filter aberrant features and those that are not correlated to the targets.

### Prediction Model Performance

**Figure 4:**
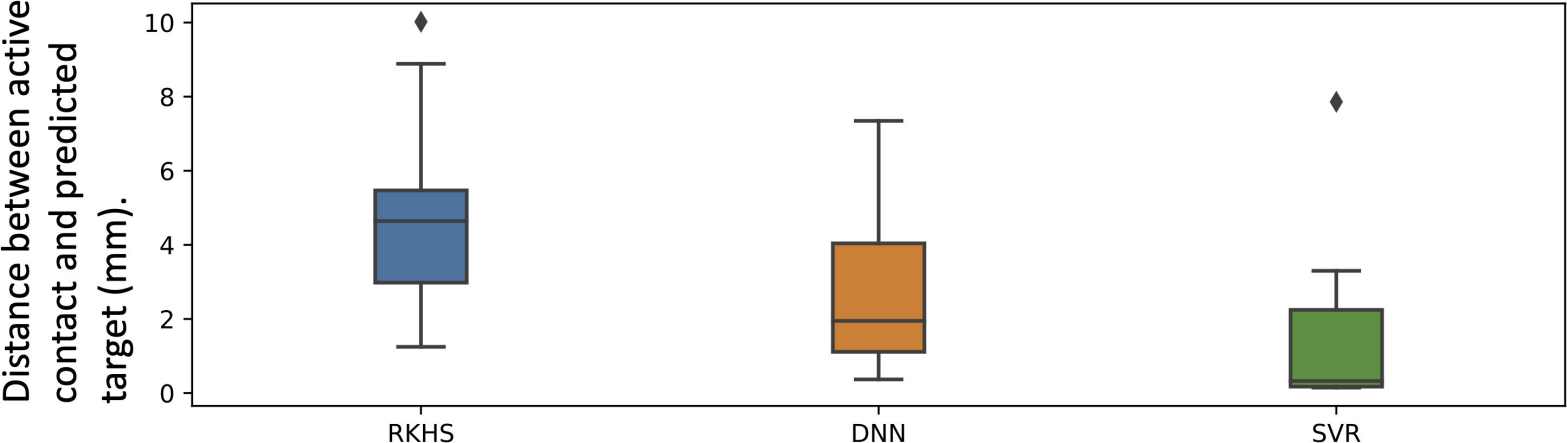
Box plot of the vector error between predicted target and active target for the three methods. RKHS method (blue box), DNN method (orange box), and SVR method (green box).

**Figure 5:**
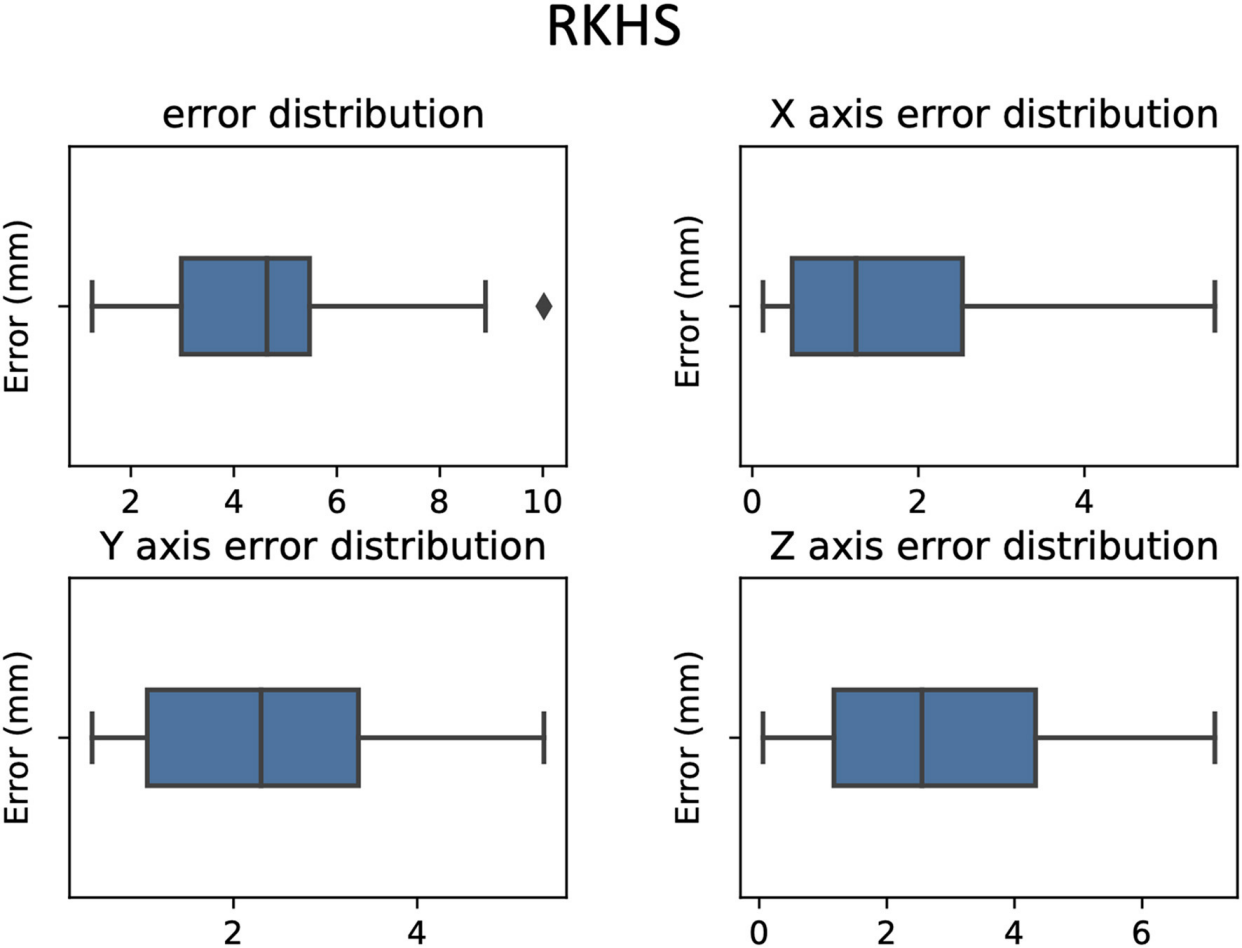
Errors between predicted target and active contact box-plot in 3D and according to each axis, RKHS model.

**Figure 6:**
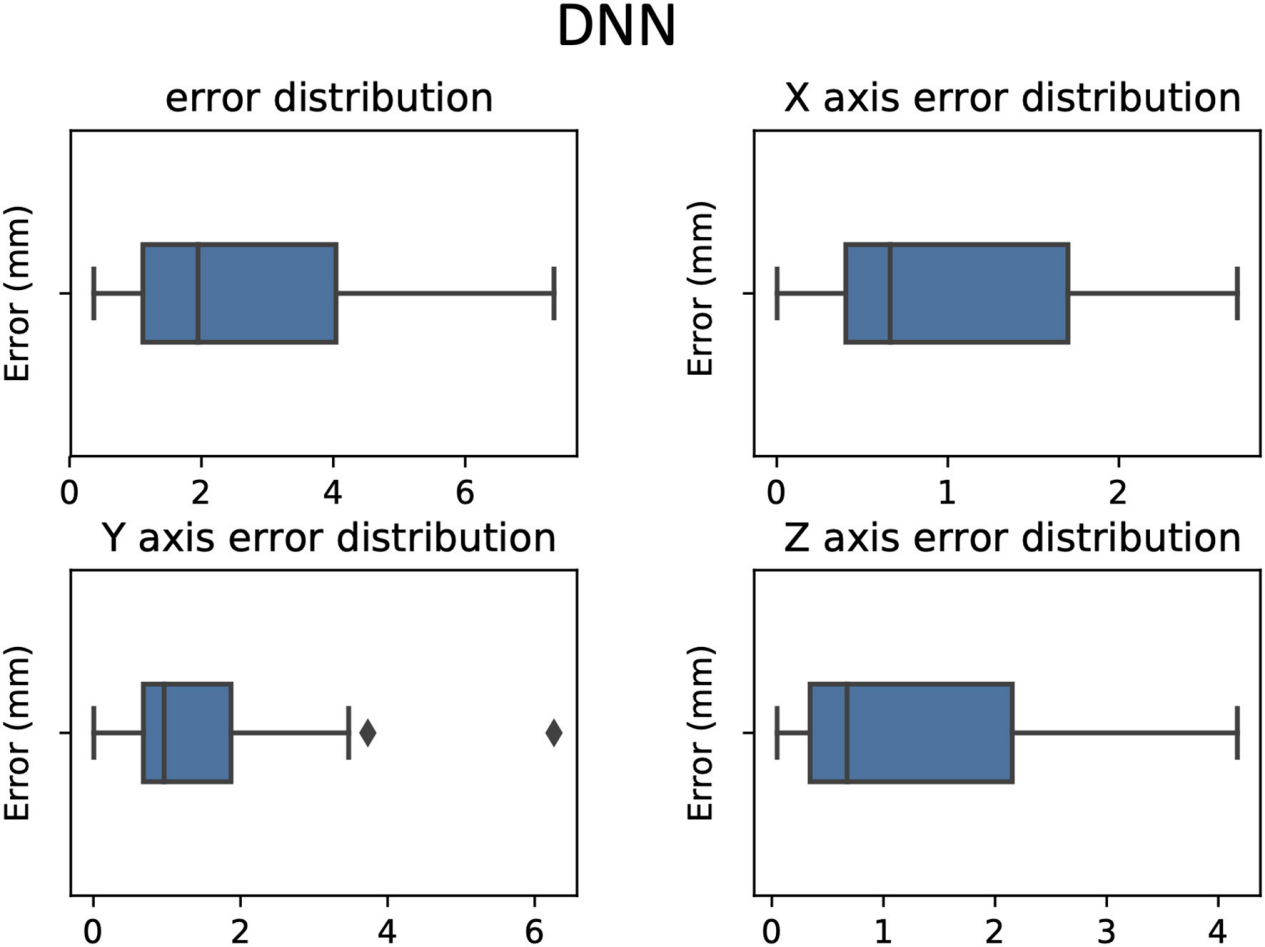
Errors between predicted target and active contact box plot in 3D and according to each axis, DNN model.

**Figure 7:**
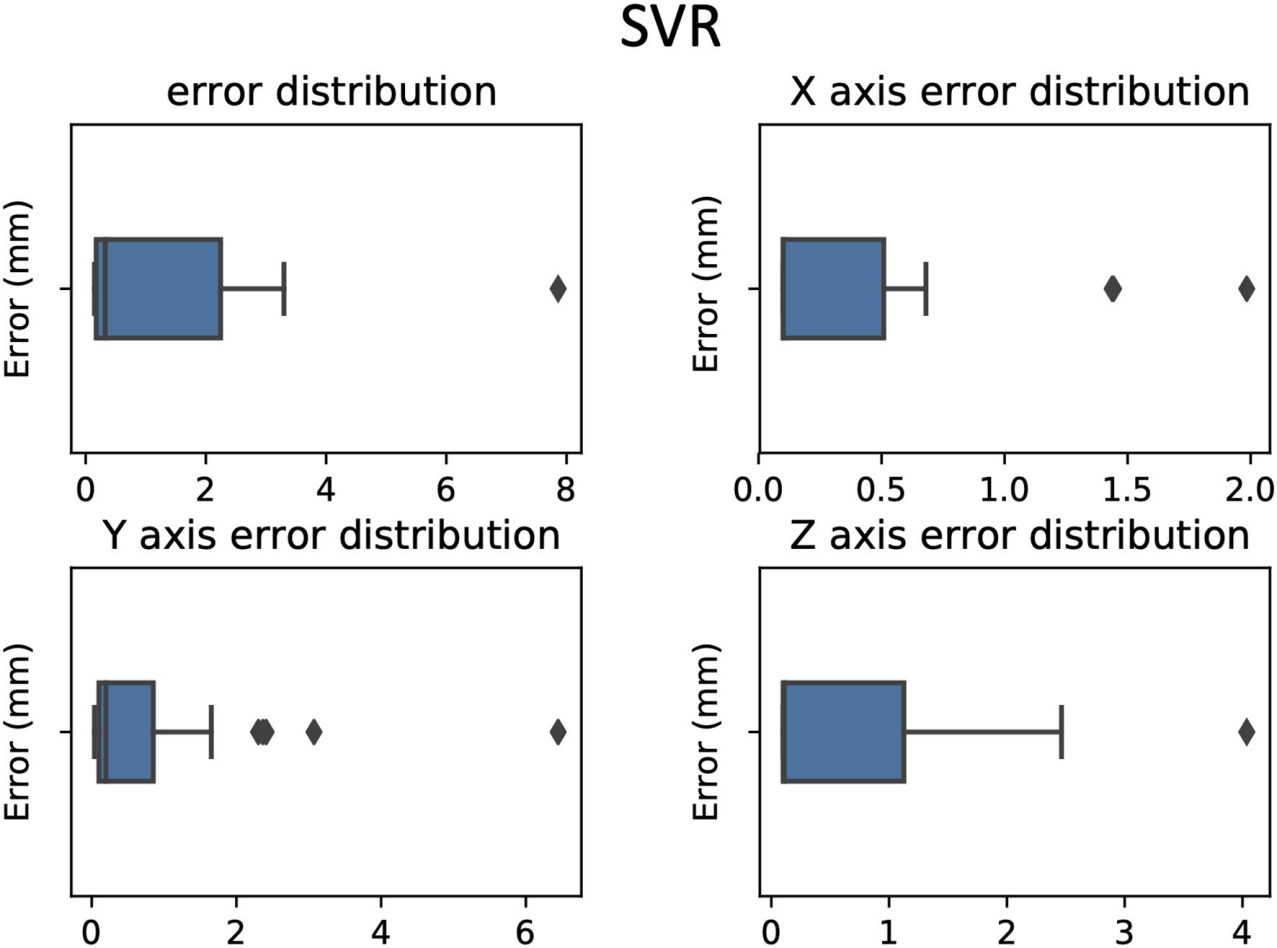
Errors between predicted target and active contact box plot in 3D and according to each axis, SVR model.

The mean and standard deviation of the distance between the predicted targets and the clinical target were as follows (mean ± standard deviation in millimeters): RKHS (4.43 ± 2.24), DNN (2.51 ± 1.69), and SVR (1.33 ± 1.64). We observed that SVR performs better than RKHS and DNN, both in terms of mean error and standard deviation as shown in Figures 4–7. SVR prediction outperformed both DNN and RKHS on each of the axes.

### Data Augmentation Effect on DNN and SVR Performance and Prediction 95% Confidence Interval

**Figure 8:**
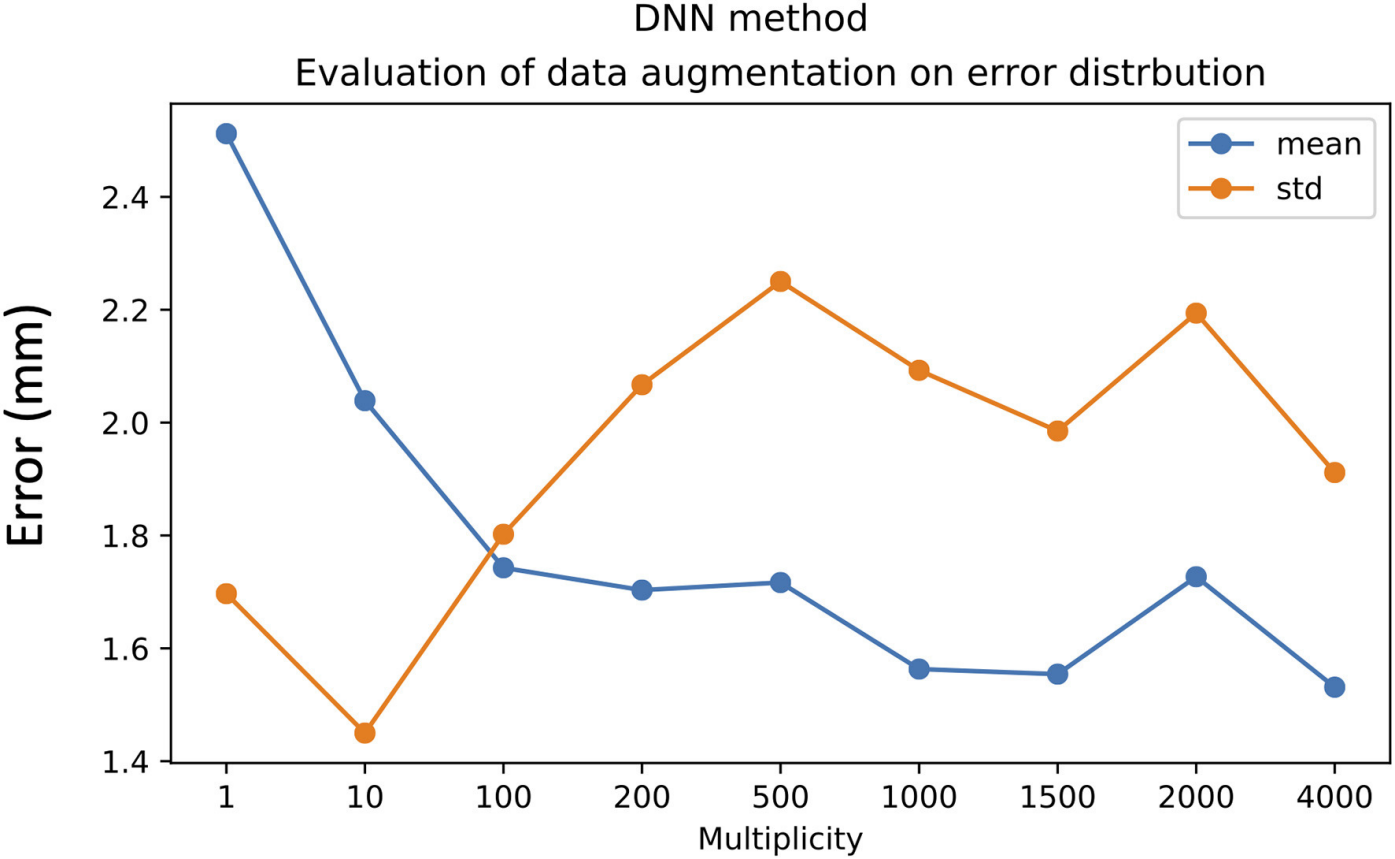
Effect of data augmentation on DNN method performance. Mean value (blue line) and standard deviation (orange line).

**Figure 9:**
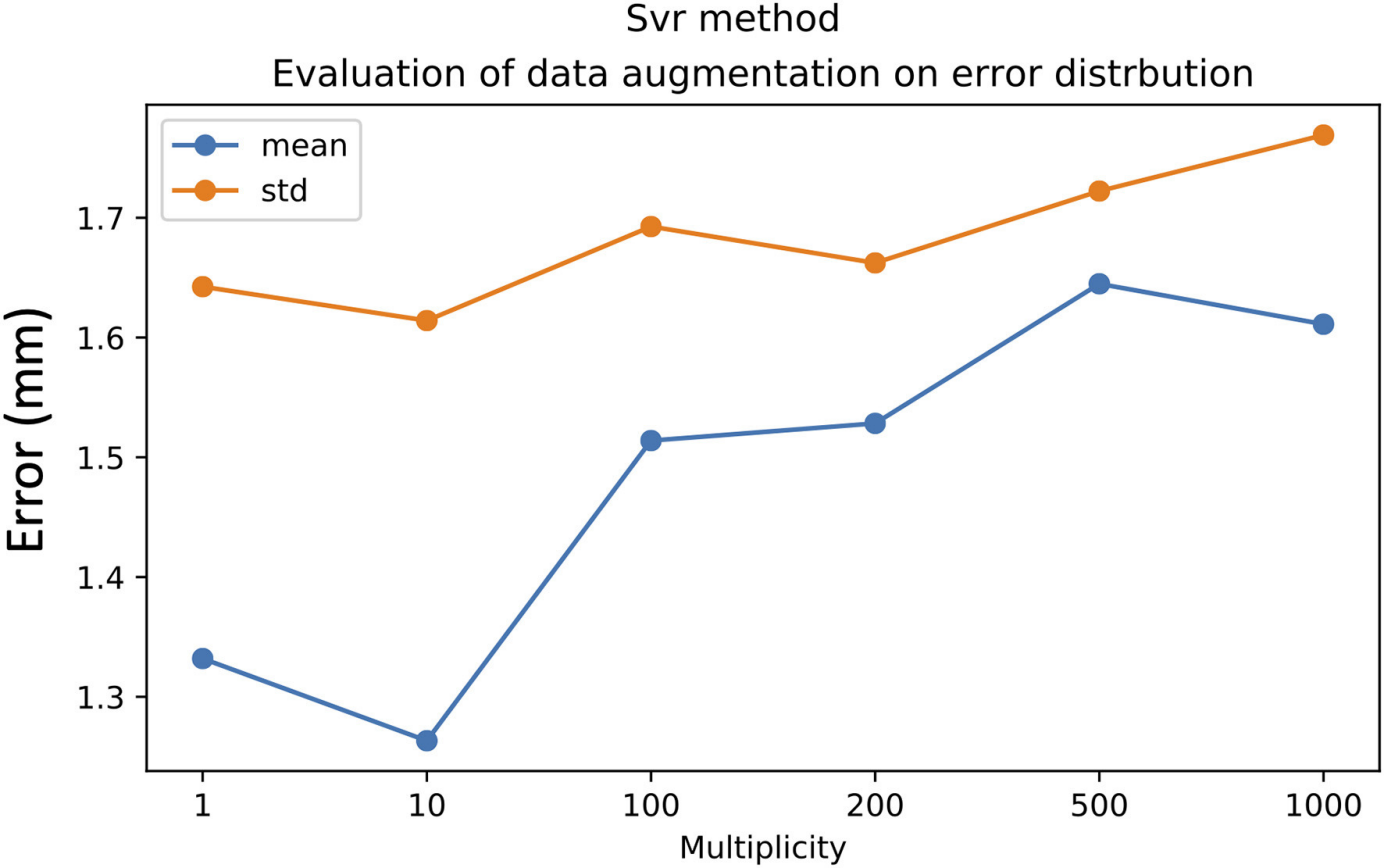
Effect of data augmentation on SVR method performance: mean value (blue line) and standard deviation (orange line).

Each database input sample was augmented by adding random noise in the range of the inter-operator variability. We showed that for the DNN method, the optimum multiplicity factor value was 100. This multiplicity value provided a good compromise between the mean value and the standard deviation; while for the SVR method, the optimum values of both mean and standard deviation were achieved with an augmentation factor of 10, as shown in Figures 8, 9.

Given the intra-observer variability in landmark positioning, the mean values of the confidence interval of the predicted target were equal to 0.5 mm for the DNN method and 0.35 mm for the SVR method (Supplementary Material).

### Predicted Target Locations on the DISTAL Atlas

**Figure 10:**
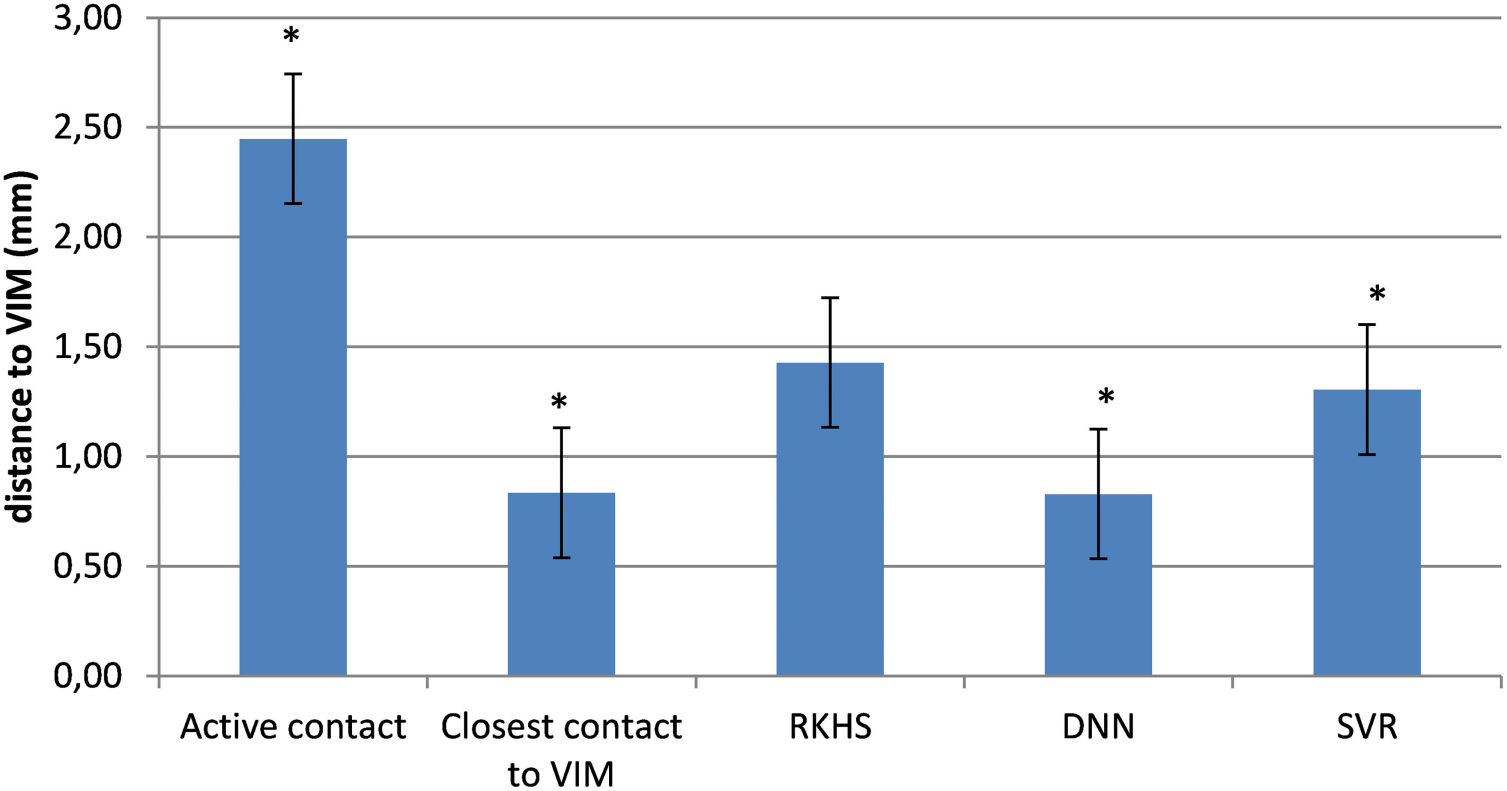
Minimum distances between targets and Vim (average histogram with standard error bars). *p < 0.05; Student's test.

**Figure 11:**
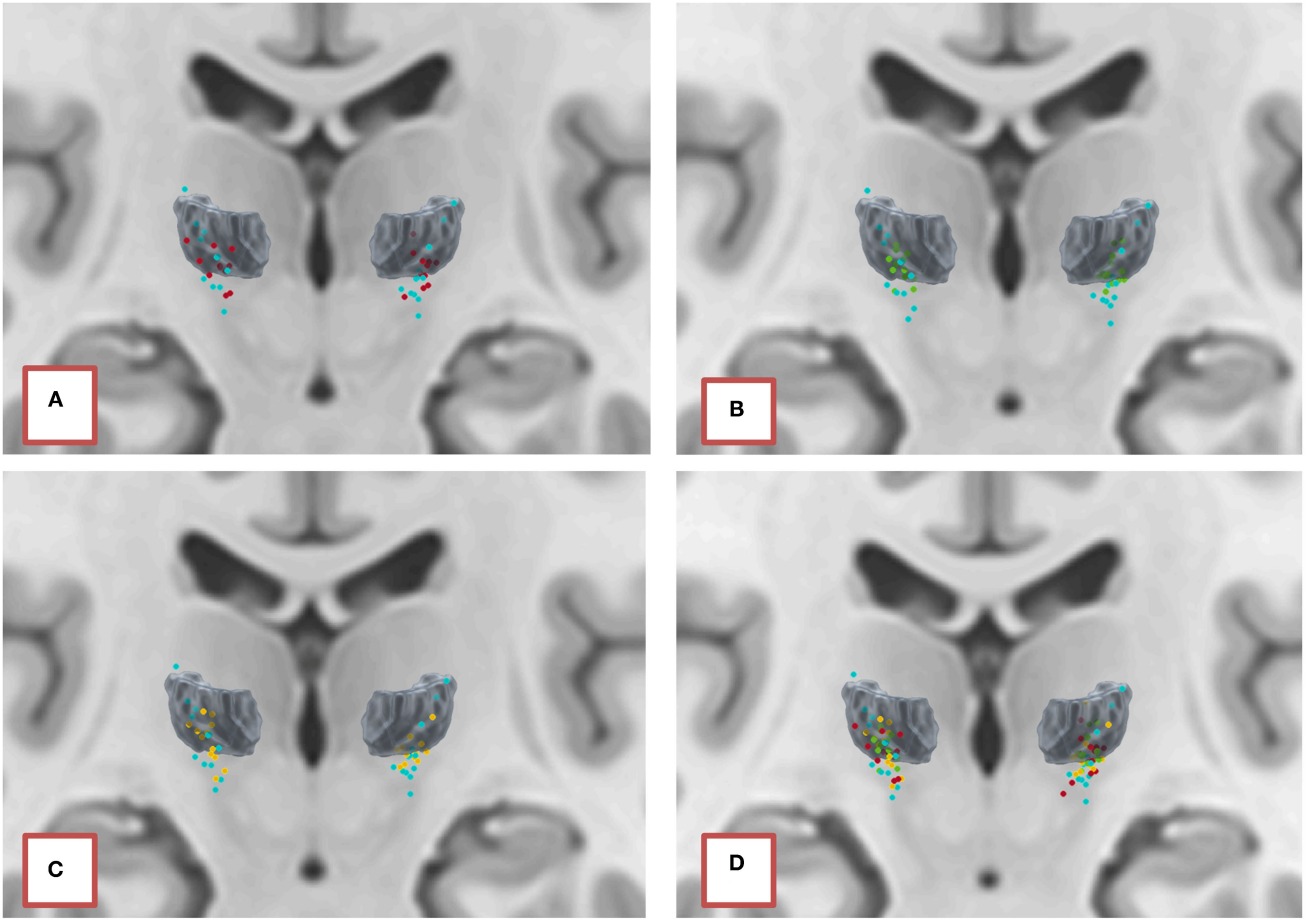
Positioning in the ICBM2009b template of active contacts (blue) and targets predicted by the models **(A)** RKHS (red), **(B)** DNN (green), and **(C)** SVR (yellow), compared to the VIM segmented according to the DISTAL atlas. **(D)** Summary of all predictions.

Figure 10 shows the distance between the DBS-lead contacts or the predicted targets (calculated according to the same LOOCV external validation procedure) on one hand and the Vim structure extracted from the DISTAL atlas in MNI space on the other hand. Figure 11 shows the spatial distribution of the 29 targets in MNI space with the VIM structure extracted from the DISTAL atlas. We first showed that the active contacts were not necessarily those closest to the VIM as delimited on the DISTAL atlas (average distance to the VIM of 2.45 for active contacts vs. 0.83 mm for the closest contact, *p* = 0.004). Secondly, we observed that the RKHS, DNN and SVR methods placed their targets fairly close to the anatomical VIM (distance <1.5 mm in average).

### Clinical Outcome in the “Validation Set” Cohort

The metamodel used for the targeting in this cohort was the RKHS model. Outcomes are reported in Table 2. The median improvement between pre- and post-operative FTM scores was 64% (IQR: 40–75) with a minimum of 0% and a maximum of 81%. The mean improvement rate was 52 ± 28%. Four patients out of 9 had an optimal outcome (improvement rate >66%). Two patients out of 9 had a failure of the surgery (improvement rate <33%). One patient had a slight improvement (20%, *d*_1_ = 2.66 m and *d*_2_ = 2.08 mm on the left side and *d*_1_ = 1.84 mm and *d*_2_ = 0.86 mm on the right side) because of the relapse of the tremor after a 3-month period of improvement, associated with the development of an ataxia. One patient had no improvement (0%) after an initial good result with a thalamotomy effect. The two DBS leads are quite far from the planned target (*d*_1_ = 5.35 mm, *d*_2_ = 3.81 mm on the left side; *d*_1_ = 12.47 mm, *d*_2_ = 8.54 mm on the right side) but the patient, who is 79 years old, does not want another intervention.

## Discussion

In this study, we aimed to define an effective target for essential tremor, not based on anatomical data, but rather on post-operative clinical outcome. We sought to predict the position of this functional target, called “Vim,” with reference to the conventionally stimulated anatomical structure, according to the radiological characteristics of a given patient. For this, we used and optimized various methods of machine learning, from a database including, on the one hand, the position of 18 landmarks per hemisphere in 15 patients (29 electrodes), and on the other hand, the position of the DBS-lead contacts whose activation led to an optimal clinical result in these same patients. We were able to predict the position of the functional target with a minimum mean error of 1.33 ± 1.64 mm. Moreover, all the models studied positioned the predicted targets at an average distance of <1.5 mm from the anatomical Vim. The database was constructed exclusively with data from patients with optimal post-operative outcome, for the reason that there is no way of knowing, for patients whose result is suboptimal or poor, if the reason is due to poor electrode positioning or to true physiological tolerance to the anti-tremor effect of thalamic DBS (non-responder patients) as explained by Papavassiliou et al. (20).

Regarding the 18 landmarks, no intra- or inter-observer error >2 mm was observed, except for the *X* coordinate of the BPT (4.14 mm between two observers) certainly linked to an overly vague definition of this landmark. Most regression models, however, especially in the feature selection stage, eliminate from the outset the reference points whose variability is outliers. Furthermore, we created the “Optim-DBS” software to calculate the target “Vim” from the metamodels developed in this study, by manually positioning the 18 landmarks. It is intended to automate the step of selecting landmarks to minimize landmark positioning variability. At present, the procedure (from loading the patient's imaging to export of the imaging marked with the predicted target) takes from 5 to 10 min on average (a video is available on the internet public page https://www.youtube.com/watch?v=Hlf7edlWhHg).

Our learning approach implied that our database should theoretically satisfy two requirements: (1) to contain the largest number of different examples (i.e., as many patients as possible) and (2) to contain the largest possible number of representations of each characteristic of the data. The data augmentation technique applied to the DNN and SVR models made it possible to meet this second requirement. We have seen that this improved the performance of these two models.

Our database may not, however, have met the first requirement, hence underlining the need to include many more patients to refine the models, and thus to set up distributed registers between the various DBS centers. Indeed, low sample size is a major issue and is explained by the relative low number of surgical procedures for essential tremor and the strict inclusion criteria in our study.

Since the number of patients included in the database was 15, our initially preferred method was RKHS, which is suitable for small databases. Since the inter-operator variability allowed us to generate artificial data, however, the DNN and SVR methods became more suitable and performed “better” than RKHS in terms of prediction error.

From a mathematical standpoint, regarding the expression of the prediction function **F** in the three cases; RKHS and SVR rely on the Euclidian distances between landmarks. This means that the closer the landmarks are from the training data, the greater the probability that the target will be close. The SVR method selects some support vectors (some hemispheres of some patients) that will be used in the expression of the prediction function. It might be counter-intuitive, but the only explanation why the SVR methods performs better than the RKHS method is the fact that the selected support vectors might be those which have a coarse representation of anatomical variability between patients. Indeed, the kernel ridge regression (RKHS) method could correspond to a particular case of the SVR method in which all the hemispheres are used as support vectors in the prediction function. The performance of the DNN method is much more difficult to explain since there is no clear (in the expression of F) causality between proximity in the landmarks space and proximity of active contact positions. Despite the lack of evidence of causality in DNN, the filters that are included in the DNN network layers allow good performance to be achieved. The DNN method, however, generally requires a higher data size, as can be observed in Figure 8. We expect that DNN would provide better performance with a larger dataset size, considering the variability of patient brain anatomy.

An initial validation step in the modeling process was cross-validation, intended to give an idea of model robustness. Given the small number of data, we opted for a leave-one-out cross-validation (LOOCV) method rather than k-folds in order to keep enough data for the training set. This method had the disadvantage, however, of forming training sets very similar to each other, and test sets very different from each other. Thus, one obtained almost the same model on each “fold” (part of N-1 data) with highly variable prediction quality according to the validation set (1 random data). It was therefore difficult to draw a formal conclusion about the calculated average error. Furthermore, the Euclidian error must be interpreted with caution because many parameters are involved: (1) the dimensions of the DBS lead (MEDTRONIC 3,389 in our study: diameter 1.28 mm and contact length: 1.5 mm) and the electric field distribution around the lead (volume of tissue activated, VTA) explain that the minimum error impacting clinical outcome is generally considered to be 2 mm (20); and (2) the lead location in stereotactic space is subject to potential measurement errors. The reconstruction of the DBS lead was based on the artifact created by the lead on the CT scan or MRI depending on the post-operative imaging in our study. On CT scan, it is known that this artifact measures approximately 3.4 mm, which is sufficiently wide to introduce errors into the calculation of the coordinates, although it is generally accepted that the axis of the electrode corresponds to the center of the metallic artifact (37). On MRI, the ferromagnetic properties of the DBS lead may contribute to creating field in homogeneities and cause image distortion. Indeed, it has been shown that the axis of the lead is slightly eccentric within the signal void, which can be as wide as 3.56 ± 0.3 mm (38), resulting in errors in the reconstruction of the electrode in stereotactic space. Thus, the mean targeting error of 1.3 ± 1.6 mm (SVR method) is (1) on average less than the minimum error impacting clinical outcome and (2) in the same range as the measurement error of the lead location on post-operative imaging.

The interpretation of vector errors between the position of the active contact and the predicted target is not straightforward. Indeed, when normalizing the active contacts in MNI space, we showed that the “functional volume” of the target was very extensive and possibly included at least two different anatomical structures (the cerebellar territory of the motor thalamus: Vim and Voi nuclei, and the subthalamic area), which is to say, from a mathematical point of view, that there was an infinity of solutions to our problem. Several authors have described the relationship between lead location and outcome. Some have described their results in the (ACPC) space mainly with data normalization according to [ACPC] distance and width of the third ventricle (19, 20, 22, 23, 39), others using probabilistic functional atlases (12), and finally others like us with normalization in MNI space (40). It emerges that there are two groups of targets (41): the “cerebellar thalamus,” including the Vim/Voi/Vop nuclei of Hassler and the posterior subthalamic area/caudal zona incerta, including the dento-rubro-thalamic tract. Interestingly, these two groups were also represented in our findings because they are part of the same neural network. A prospective randomized cross-over double-blind trial for patients with essential tremor showed that DBS of the PSA is not significantly different from DBS of the Vim in suppressing tremor, but clinical benefit from PSA-DBS is achieved at lower stimulation amplitudes (42).

As the Vim nucleus was the anatomical structure theoretically targeted under electrophysiological guidance in our cohort of patients, we wanted to calculate the distance between the predicted targets and this structure. Theoretically, three methods could have been used.

The first one, used in numerous studies on the position of active contacts (23), or to validate targeting methods (43), consists in correlating the final position of the implanted electrodes, or the pre-operative target, with the intra-operative coordinates of the target given by the micro-electrode recordings. The AC-PC stereotactic referential of a given patient, however, is uncontrollably deforming once the first burr hole has been made due to brain-shift. Indeed, it is widely debated whether any comparison, between the AC-PC coordinates of a pre-operative or post-operative target, with intra-operative AC′-PC′ coordinates of the electrophysiologically defined structure during the procedure, may be significantly biased (44, 45).

The second, theoretically, the most accurate, would have been to position the predicted target on a WMn sequence acquired at 3 or even 7 T, allowing the intra-thalamic nuclei (7, 46) to be directly visualized. Such images, however, could not be acquired in already implanted patients. This sequence, available in our institution, is now used prospectively for all patients who are candidates for Vim-DBS. The comparison between the position of the Vim on WMn imaging with that of the predicted functional target and the definitive position of the active contact will be the subject of a future study.

The third approach, the one we chose, was to position the targets on an atlas [DISTAL atlas (11)]. In this approach, we normalized the predicted targets in the ICBM 2009b template (MNI) and calculated the minimum distance between this prediction and the Vim as segmented by the atlas. Similarly, we calculated the distance between each DBS lead contact and the VIM. First, we observed that the active contacts were not necessarily those closest to the VIM (average distance to the VIM of 2.45 mm for the active contact vs. 0.83 mm for the closest contact, *p* = 0.004). This result could be explained either by the fact that there was an error in atlas normalization and/or registration method, that the atlas itself was inaccurate or that the best clinical target was not necessarily in the anatomical Vim (recall that nearly half of the active contacts in our study were located under the thalamus according to the atlas). Secondly, we showed that the predicted targets, whatever the model, were on average <1.5 mm from the VIM, which nevertheless conferred a degree of confidence in the performance of the models.

By showing, however, that active contacts (thus “real” clinically effective targets) are not necessarily those closest to VIM, reflecting a degree of anatomo-functional discordance (which may be real or artifactual due to the use of atlases), we illustrated the fact that a simple anatomical validation (whether radiological, electrophysiological, or atlas-based) is insufficient to prejudge the validity of our modeling. The only way to validate this target is to be able to test it in the operating room and to compare the clinical results obtained when the electrode is implanted in the predicted target to that obtained by the conventional method. With this objective, we have initiated a multi-center prospective study (Opti-VIM study, NCT03760406).

To illustrate the potential clinical interest of this targeting method, we provided the first results of a cohort of consecutive patients operated on under general anesthesia without MER with the RKHS metamodel (using the OptimDBS software) and not included in the Opti-VIM trial. This sample of patients is particular because of their age and comorbidities (mainly cerebral atrophy) that precluded for awake surgery with MER and clinical testing and thus was an exclusion criterion for the Opti-VIM trial. We offered the possibility of an asleep surgery without electrophysiology as an “off-label” targeting procedure and 9 patients were operated on. We chose the RKHS model because it was the first developed in the OptimDBS software and used at the beginning of the Opti-VIM clinical trial. As the model cannot be changed during the study, which is still running with this model, we also used this model to operate on the patients outside the trial.

The clinical results of this case series, with a median improvement rate in the FTM scores between pre- and post-operative assessments of 64% (IQR 40–75) at a mean follow-up of 14 months (±9), are within the same range as the improvement rates compiled in a recent literature review (47) which were between 66 and 78% at 1 year post-operatively for bilateral implantation under local anesthesia with MER.

To the best of our knowledge, this study is the first one to attempt to retrieve a DBS target based on the patient's own anatomy and post-operative outcomes of previous patients using machine learning algorithms. Our method is part of an approach described by Talairach in the 1950s for thalamotomy procedures, intended to retrieve anatomical structures invisible by ventriculography, according to the position of other structures which were visible (mainly the ventricles). Several authors then described the average position, in the (AC-PC) space, of the active contacts of the DBS leads implanted in patients with good post-operative outcomes. For example, Table 3 gives examples of statistical targets for the Vim.

**Table 3:** Published statistical targets for DBS in essential tremor.

**References**	***X***	***Y***	***Z***
Benabid et al. (1)	11.5 + V3	1/12 × [ACPC]	0
Blond et al. (14)	15	6	+1
Ondo et al. (49)	12–14	−4 to 5 (MCP)	+1
Benabid et al. (50)	15.36	0.296 × [CA-CP]	0
Mobin et al. (17)	11.2	5.38	0
Koller et al. (39)	11.5 + V3	1/12 [CACP] ×2.5	0
Papavassiliou et al. (20)	12.3	6.3	−1
Hamel et al. (22)	12.7	−7 (MCP)	−1.5
Sandvik et al. (41)			
VIM	13	−1.8 (MCP)	4.1
PSA	12	−5.8 (MCP)	−1.5
Chen et al. (4)	10.5 + V3	−0.25 × [ACPC] (MCP)	0

*All coordinates are given in millimeters from PC (posterior commissure); [ACPC]: distance between AC (anterior commissure) and PC; (MCP): distance in mm from the mid-commissural point (i.e., the middle of [ACPC]); V3: width of third ventricle/2 in millimeters. VIM, ventro-intermedius nucleus of the thalamus; PSA, posterior subthalamic area*.

In Table 3, we note that correction of the lateral coordinate (X) according to the width of the third ventricle (V3) is rarely carried out, just as the Z coordinate is never normalized. The anteroposterior coordinate (Y) is often expressed according to the [AC-PC] distance. On the one hand, Talairach had clearly shown from the start of stereotaxic studies in humans that such coordinates, summarized in a simple arithmetic mean, cannot be used to determine the position of a target in a given patient (48). On the other hand, Chen et al. (4, 5), using “standard” statistical targeting (see Table 3), showed in a retrospective study followed by a prospective non-randomized study that post-operative improvement was not statistically different between patients operated under general anesthesia without electrophysiology and clinical testing and those operated on with the standard awake procedure. From a theoretical standpoint, however, this “statistical” targeting has two main limitations. First, the anatomical basis of the linear relationship between the positions of internal structures of the brain is hazardous. Secondly, this approach only seeks to find a single landmark on each plane of the stereotactic space. Incorporating multiple landmarks into the modeling of the target position, however, should result in a more robust model. Thus, our approach differs by the multiplicity of landmarks and non-linear modeling.

The strength of our study is that it describes an original evolving targeting method for DBS in essential tremor based on the patient's own imaging on a native image. This implies great material simplicity as only a 3D T1-weighted imaging at 1.5 Tesla is needed and there is no need for image post-processing (no normalization/atlas co-registration/DTI processing, etc.) which could be a potential source of targeting errors. Furthermore, the landmarks used in our method can be localized with a very high degree of reproducibility and the models are robust with regard to any placement error that could be made by the surgeon. Finally, this is an evolving approach (artificial intelligence) because the models are supposed to improve as they are used with more patients included in the learning sets.

However, all these results must be interpreted with caution given the small number of subjects used in the training set and the lack of external validation on a large number of patients. We recall that the validation of this targeting model will be provided by the clinical results (efficacy, side effects) of the prospective multicentre Opti-VIM trial.

## Conclusion

In this study, using machine-learning algorithms, we predicted a clinical target for DBS in essential tremor. The learning database consisted of clinical and radiological features of patients previously operated on with optimal outcome. A prospective clinical trial (Opti-VIM) is now ongoing to validate this approach. Other perspectives include automatic landmark localization by the machine and the creation of a multi-center registry to enlarge the learning database. This would allow more variables to be included in the models, such as patient and disease characteristics, brain MRI data (e.g., functional connectivity), more post-operative data (side effects, stimulation parameters and suboptimal outcomes), etc., to optimize the models and make classifications to study whether different models can apply to different patient groups with different targets.

## Data Availability Statement

The data analyzed in this study was subject to the following licenses/restrictions: Pre and Post DBS surgery MRI data could not be shared as we can identify patients from this data. Requests to access these datasets should be directed to emmanuel.cuny@chu-bordeaux.fr.

## Ethics Statement

The studies involving human participants were reviewed and approved by CPP OUEST IV (Comité de Protection des Personnes Ouest IV), CHU de Nantes France. The patients/participants provided their written informed consent to participate in this study.

## Author Contributions

Research project: JE, EC, NZ, DG, and OB: conception. JE, EC, NZ, DG, PB, ND-P, and OB: organization. All authors: execution. Mathematical and statistical analysis: JE and NZ: design. JE, NZ, NG, and L-AS: execution. All authors: review and critique. Manuscript: JE, NZ, and NG: writing of the first draft. All authors: review and critique. Clinical data analysis: JE, DG, PB, ND-P, CD-L, and JT.

## Conflict of Interest

The OptimDBS software that was used in this study was transferred to the startup Rebrain in which EC and NZ are shareholders. The remaining authors declare that the research was conducted in the absence of any commercial or financial relationships that could be construed as a potential conflict of interest.

## Publisher's Note

All claims expressed in this article are solely those of the authors and do not necessarily represent those of their affiliated organizations, or those of the publisher, the editors and the reviewers. Any product that may be evaluated in this article, or claim that may be made by its manufacturer, is not guaranteed or endorsed by the publisher.
