# Supplementary material for: Prediction of Clinical Deep Brain Stimulation Target for Essential Tremor From 1.5 Tesla MRI Anatomical Landmarks
Source: Front Neurol. 2021 Oct 27;12:620360. doi: 10.3389/fneur.2021.620360 (PMC8579860; doi:10.3389/fneur.2021.620360)
Supplement: Supplementary file 1 [file Data_Sheet_1.docx]

Supplementary material

# Prediction of Clinical Deep Brain Stimulation Target for Essential Tremor from 1,5 Tesla MRI anatomical Features

Julien Engelhardt^1,2^, MD, PhD; Emmanuel Cuny^1,2^, MD; Dominique Guehl^2,3^, MD, PhD; Pierre Burbaud^2,3^, MD, PhD; Nathalie Damon-Perrière^2,3^, MD; Camille Dallies Labourdette^2,3^; Juliette Thomas^2,3^, MD; Olivier Branchard^1^, MSc; Louise-Amélie Schmitt^4^, PhD; Narimane Gassa^4^, MSc; Nejib Zemzemi^4,5^, PhD.

Affiliations:

1. : Department of Neurosurgery, University Hospital of Bordeaux, France
2. : Institute for Neurodegenerative disorders, CNRS – University of Bordeaux, France
3. : Department of Neurology, University Hospital of Bordeaux, France
4. : INRIA Bordeaux Sud-Ouest Research Centre, France
5. : Mathematical Institute of Bordeaux, University of Bordeaux, France

Corresponding author:

Dr. Julien Engelhardt

Service de Neurochirurgie B. Hôpital Pellegrin. Place Amélie Raba-Léon. 33076 Bordeaux Cedex. France.

Phone : +33556795679

e-mail : [julien.engelhardt@gmail.com](mailto:julien.engelhardt@gmail.com)

# Supplementary material: tables

Table I. Anatomical localization of active contacts on the DISTAL atlas in the ICBM2009b standardized space according to the Hassler nomenclature.

| Anatomic structure | Active contacts (%) |
| --- | --- |
| Zce | 1 (3%) |
| Vim | 8 (28%) |
| Voi | 3 (10%) |
| ZI | 14 (48%) |
| Aberrant position (contacts excluded) | 3 (10%) |

*Zce : zentrolateralis caudalis pars externa ; Voi : Ventro-oralis internus ; Vim : Ventro-intermedius ; ZI : zona incerta*

Table II. Intra and inter-observer variability for landmarks and target

| Landmark | Axis | Mean intra-obs. Variability (mm) | Mean inter-obs. Variability (mm) | Corresponding number for figures 5 and 6 |
| --- | --- | --- | --- | --- |
| AC | X | 0,00 | 0,00 | 1 |
|  | Y | 0,41 | 0,23 | 2 |
|  | Z | 0,00 | 0,00 | 3 |
| A | X | 0,28 | 0,39 | 4 |
|  | Y | 0,20 | 0,11 | 5 |
|  | Z | 0,00 | 0,00 | 6 |
| B | X | 0,28 | 0,39 | 7 |
|  | Y | 0,20 | 0,11 | 8 |
|  | Z | 0,52 | 0,79 | 9 |
| C | X | 0,28 | 0,39 | 10 |
|  | Y | 0,20 | 0,11 | 11 |
|  | Z | 0,26 | 0,39 | 12 |
| D | X | 0,28 | 0,39 | 13 |
|  | Y | 0,74 | 0,46 | 14 |
|  | Z | 0,26 | 0,39 | 15 |
| PA1 | X | 0,46 | 0,44 | 16 |
|  | Y | 0,49 | 0,53 | 17 |
|  | Z | 0,26 | 0,39 | 18 |
| PM1 | X | 0,69 | 0,61 | 19 |
|  | Y | 0,77 | 0,74 | 20 |
|  | Z | 0,26 | 0,39 | 21 |
| PP1 | X | 0,80 | 0,88 | 22 |
|  | Y | 1,30 | 0,93 | 23 |
|  | Z | 0,26 | 0,39 | 24 |
| BAT | X | 1,05 | 1,42 | 25 |
|  | Y | 0,65 | 0,69 | 26 |
|  | Z | 0,26 | 0,39 | 27 |
| BPT | X | 1,70 | 4,14 | 28 |
|  | Y | 0,56 | 1,36 | 29 |
|  | Z | 0,26 | 0,39 | 30 |
| CH | X | 0,39 | 0,42 | 31 |
|  | Y | 0,38 | 0,37 | 32 |
|  | Z | 0,26 | 0,39 | 33 |
| PA2 | X | 0,44 | 0,47 | 34 |
|  | Y | 1,08 | 0,65 | 35 |
|  | Z | 0,26 | 0,39 | 36 |
| PM2 | X | 0,52 | 0,45 | 37 |
|  | Y | 0,92 | 0,93 | 38 |
|  | Z | 0,26 | 0,39 | 39 |
| PP2 | X | 0,44 | 0,87 | 40 |
|  | Y | 1,28 | 1,10 | 41 |
|  | Z | 0,26 | 0,39 | 42 |
| PA3 | X | 0,56 | 0,66 | 43 |
|  | Y | 0,80 | 0,80 | 44 |
|  | Z | 0,26 | 0,39 | 45 |
| FMT | X | 0,43 | 0,40 | 46 |
|  | Y | 0,28 | 0,38 | 47 |
|  | Z | 0,26 | 0,39 | 48 |
| Pculm | X | 0,50 | 0,50 | 49 |
|  | Y | 0,77 | 0,74 | 50 |
|  | Z | 0,97 | 0,79 | 51 |
| Plat | X | 0,52 | 0,39 | 52 |
|  | Y | 0,77 | 0,74 | 53 |
|  | Z | 1,47 | 1,52 | 54 |
| Active contact | X | 0,26 | 0,24 |  |
|  | Y | 0,43 | 0,24 |  |
|  | Z | 0,62 | 0,34 |  |

# Supplementary material figures


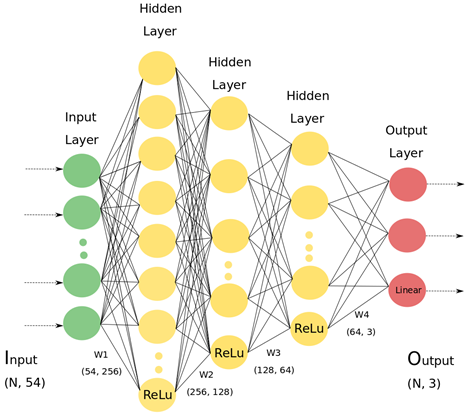


[Figure SM 1. The architecture of the deep neural network used.](#_Toc16601144)


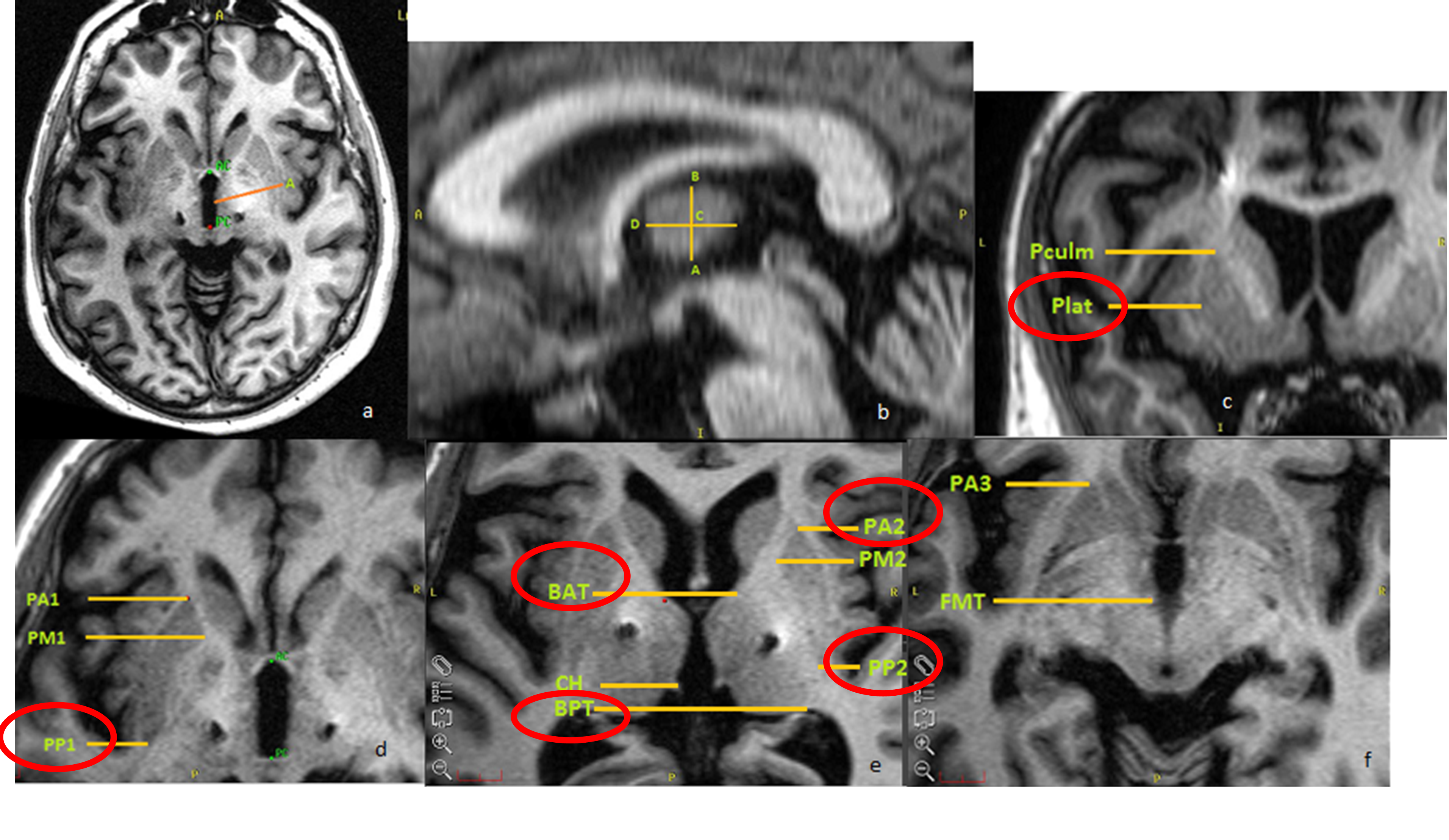


[Figure SM 2. T1 WI MRI in different planes (see figure 1). Red circle: intra and/or inter-observer variability > 1mm for at least one coordinate (x, y or z).](#_Toc16601145)


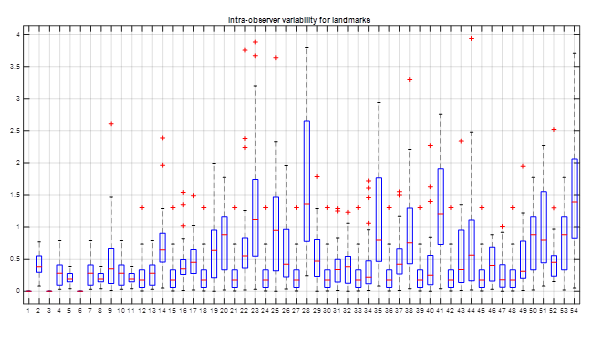


[Figure SM 3. Intra-observer variability for landmarks box-plot (mm), see table 3 for landmark-number correspondence.](#_Toc16601146)


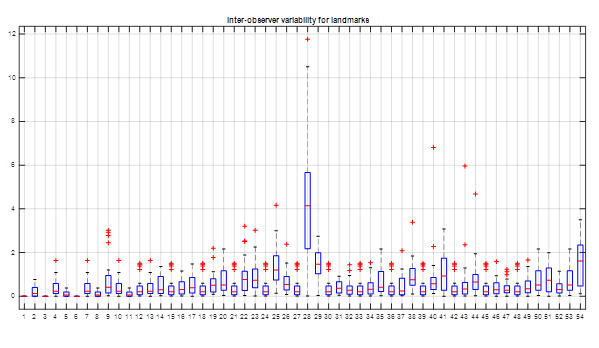


[Figure SM 4. Inter-observer variability for landmarks box-plot (mm), see table 3 for landmark-number correspondence.](#_Toc16601147)


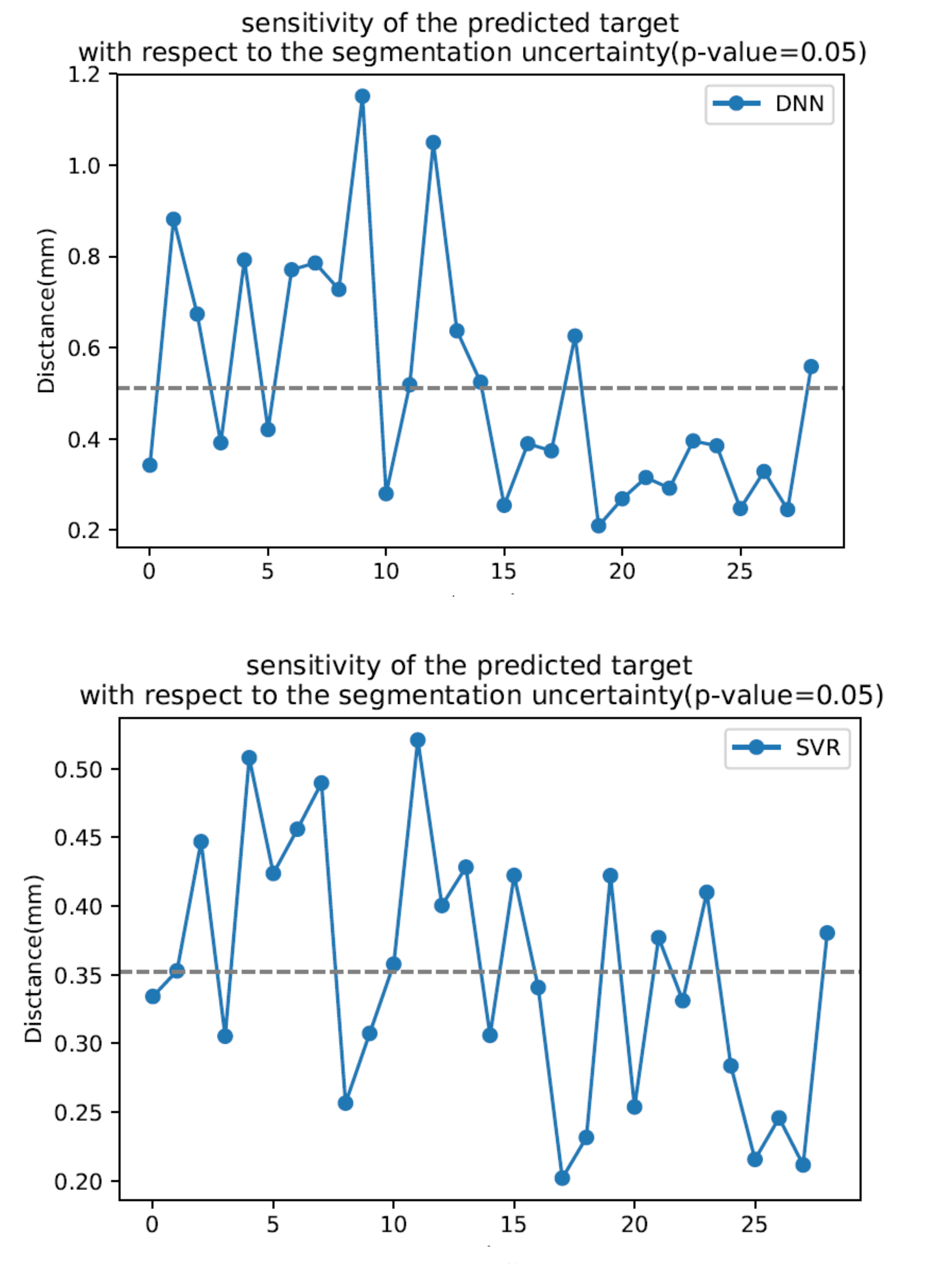


Figure SM 5. Sensitivity of the predicted target with respect to the segmentation uncertainty. The x-axis provides the patient number and the y-axis gives the radius of the confidence interval from the mean value of the predicted target.
